# Prevalence of internet gaming disorder and measurement invariance of the IGDS9-SF in a high-risk Latin American sample

**DOI:** 10.3389/fpsyt.2026.1913038

**Published:** 2026-09-10

**Authors:** John Blandon, Arnau Carmona, Bianda J. González-Santos, Jan Ivern, Marta Beranuy, Mark D. Griffiths, Xavier Carbonell, Diana X. Puerta-Cortés

**Affiliations:** 1 Social Research Institute, Institute of Education, University College London, London, United Kingdom; 2 Departamento de Psicología, Universidad de los Andes, Bogotá, Colombia; 3 Departamento de Psicología, Universidad de Ibagué, Ibagué, Colombia; 4 Institut de Recerca Sant Joan de Déu, Esplugues de Llobregat, Spain; 5 Parc Sanitari Sant Joan de Déu, Sant Boi del Llobregat, Spain; 6 Facultat de Psicologia, Universitat de Barcelona (UB), Barcelona, Spain; 7 Facultat de Psicologia, Ciències de l'Educació i de l'Esport (FPCEE) Blanquerna, Universitat Ramon Llull, Barcelona, Spain; 8 Facultad de Ciencias de la Salud, Universidad Pública de Navarra, Pamplona, Spain; 9 Psychology Department, Nottingham Trent University, Nottingham, United Kingdom

**Keywords:** behavioral addictions, IGDS9-SF, internet gaming disorder, Latin America, prevalence, video games

## Abstract

**Introduction:**

The present study examined the psychometric properties of the nine-item Internet Gaming Disorder Scale-Short Form (IGDS9-SF) in five Latin American countries. The study aimed to: (i) estimate internet gaming disorder (IGD) prevalence; (ii) test cross-national measurement invariance in a high-risk sample; and (iii) evaluate construct validity by testing the impact of removing Item 8 (escapism) in this population.

**Methods:**

A total of 10,151 *League of Legends* players aged 18 to 35 years (19.9% female; *M*_age_ ​ = 20.84 years, *SD* = 2.9) from Mexico, Colombia, Peru, Chile, and Ecuador completed the IGDS9-SF. General population prevalence was estimated via a weighted estimator. A probable IGD subsample (n = 1,248; 20.1% female; *M*_age_ = 20.3 years, *SD* = 2.9) was used for multigroup confirmatory factor analysis.

**Results:**

The observed rate of probable IGD among recruited players was 12.3%; an exploratory extrapolation to the general population (51.38% regional gamer proportion) yielded 6.32% (95% CI [5.99, 6.65]), with national rates ranging from 4.88% (Chile) to 8.27% (Ecuador). In the probable IGD subsample, metric invariance was supported across countries, whereas scalar and strict invariance were not established. A one-factor model excluding Item 8 and freeing the residual correlation between Items 2 and 3 provided a significantly better structural fit than the original nine-item model. Strict invariance was supported across sex and server. For preferred game mode, metric invariance was supported, but scalar and strict invariance were not established.

**Conclusions:**

The IGDS9-SF appears to be a robust instrument for assessing IGD severity among high-risk Latin American samples, supporting cross-national comparisons at the metric level, and more directly across sex and server. However, the findings identified the ‘escapism’ criterion (Item 8) as psychometrically weak, challenging its inclusion as a core DSM-5 diagnostic symptom. These results highlight the need to reconsider the conceptualization of IGD.

## 1 Introduction

In 2023, the global gamer base reached 3.31 billion people (40.9% of 8.09 billion globally), with particularly high engagement in Latin America, where 335 million of 652 million people (51.4%) are regular gamers (1–3). However, the increasing prevalence of digital media has led to the possibility that a minority of gamers will develop potentially disordered/addictive patterns of use.

Internet gaming disorder (IGD) is characterized by excessive or compulsive gaming leading to significant real-life impairment, including loss of control, tolerance, and withdrawal (4, 5). Notably, emerging adulthood represents a particularly vulnerable developmental stage for its onset (6). Among this population, IGD is consistently associated with a range of adverse outcomes, including internalizing symptoms, psychosocial dysfunction, and deficits in impulse control, a finding supported by several meta-analyses (7–9).

Significant controversy surrounds the diagnostic criteria for IGD, creating a complex challenge for its clinical identification and research assessment. This diagnostic ambiguity is a critical public health issue, given the vast reach of modern gaming. Some of this controversy centers on the discrepancies between the 11^th^ revision of the International Classification of Diseases (ICD-11) (10) and the fifth edition of the Diagnostic and Statistical Manual of Mental Disorders (DSM-5) (4) diagnostic criteria frameworks (11–14). For example, a meta-analysis reported a prevalence of 7.9% with the DSM-5 criteria, compared to only 3.0% with the ICD-11 (15). Similarly, a large global study with 123,262 gamers reported 4.97% using the DSM-5 framework versus 1.96% using the ICD-11 (16). This diagnostic discrepancy is particularly pronounced in Latin America, where reported prevalence ranges widely from 1.1% to 38.2% (17).

This diagnostic inconsistency extends to psychometric assessment. A systematic review found that while eight instruments fully covered the DSM-5 diagnostic criteria, no single instrument encompassed all three core criteria and five areas of functional impairment of the ICD-11 framework (18). Even the most widely used of these DSM-5 instruments, the Internet Gaming Disorder Scale–Short Form (IGDS9-SF) (19), has a notable limitation: although some studies have included vulnerable populations (20), its validation has primarily involved general populations, typically university students, rather than high-risk gamers (21).

Moreover, studies examining the psychometric invariance of the IGDS9-SF, whether reporting full or partial invariance, have been conducted exclusively with low-risk samples such as students or non-problematic gamers (5, 13, 22–27). Critically, the scale’s invariance remains untested in high-risk populations, a methodological gap that prevents researchers from determining whether observed differences between groups reflect true variations in the construct or are merely psychometric artifacts (28, 29).

Further complicating its measurement, the validity of Item 8 of the IGDS9-SF, which assesses gaming to escape negative moods, has recently been questioned. Drawing on the same broader data collection analyzed in the present study, a prior report examined the general population of 10,136 Latin American gamers and found that removing this item substantially improved the scale’s model fit (23). This finding aligns with previous research indicating the item has low clinical utility and fails to distinguish between problematic and non-problematic gamers (21, 30–32). This raises critical questions about the conceptualization of IGD, prompting a debate about whether the ‘escape’ motive reflects a diagnostic criterion in itself or simply a typical gaming motivation that may lead to the disorder when it becomes excessive (e.g., 23, 33).

To minimize heterogeneity, the present study focused on Multiplayer Online Battle Arena (MOBA) players, a genre structurally associated with elevated IGD risk (34–36). Consistent with this, recent psychometric work has empirically linked specific structural MOBA design features—particularly reward-related mechanics—to IGD severity in gaming populations (37). MOBA game mechanics, such as intense competition and reliance on temporary teams, can generate toxic social dynamics that reinforce problematic play patterns and elevate IGD risk (34, 38–40).

Taken together, two key psychometric issues emerge: (i) the lack of invariance data among high-risk groups and (ii) the questionable validity of a core diagnostic item (i.e., escapism). Addressing these gaps is essential for refining the IGD construct and producing more accurate epidemiological estimates. Clarifying the psychometric validity of IGDS9-SF in this context can inform future cross-national screening and intervention strategies targeting problematic gaming behavior.

Therefore, the present study was designed to systematically investigate these epidemiological and psychometric inconsistencies. More specifically, the study’s objectives were to: (i) estimate a prevalence of IGD in five Latin American countries using the original nine-item IGDS9-SF and its established cut-off; (ii) test the scale’s cross-national invariance in IGD high-risk sample; and (iii) evaluate construct validity by determining whether removing Item 8 provides a better model fit in IGD high-risk sample.

## 2 Materials and methods

### 2.1 Participants

A non-probability convenience sample of *League of Legends* (*LoL*) players was recruited. Of the initial 11,544 respondents, 978 were excluded for not meeting inclusion criteria, and 415 for providing invalid responses. These data derive from the same broader online data collection reported in Blandon et al. (23), which examined Item 8 removal in the general population of recruited players. The present study applies additional data-cleaning criteria and restricts all psychometric analyses to a distinct high-risk subsample (see Statistical Analysis). The final sample comprised 10,151 participants (20.1% female; *M*_age_ ​ = 20.84 years, *SD* = 2.9, range = 18–35 years) from Mexico (50.2%), Colombia (20.9%), Chile (12.6%), Peru (10.2%), and Ecuador (6.1%).

Using the IGDS9-SF cut-off score of ≥32 (20), a subsample of 1,248 participants with probable IGD was identified, representing 12.3% of the total sample. This subsample included 20.2% female (*M* age​ = 20.3, *SD* = 2.9) and was distributed across Mexico (48.6%, *n* = 606), Colombia (23.9%, *n* = 299), Peru (9.8%, *n* = 122), Chile (9.7%, *n* = 121), and Ecuador (8.0%, *n* = 100). The subsample with non-probable IGD consisted of 8903 players (20.0% female; *M* age = 21.0, *SD* = 3.2) and was distributed across Mexico (50.4%, *n* = 4489), Colombia (20.5%, *n* = 1821), Peru (10.3%, *n* = 915), Chile (13.0%, *n* = 1155), and Ecuador (5.9%, *n* = 523).

### 2.2 Measures

Sociodemographic Data and LoL Usage Questionnaire (self-developed). This instrument comprised 10 items to gather sociodemographic data, including age, sex, educational level, and country of residence. It also included questions specifically related to *LoL* usage, such as game server and preferred game mode.

Spanish version of the Internet Gaming Disorder Scale-Short-Form (IGDS9-SF) (23). The nine-item IGDS9-SF (19) was used to assess the nine DSM-5 criteria for IGD on a five-point Likert scale from 1 (*never*) to 5 (*very often*). Higher scores reflect greater IGD severity. In the present study, the items were adapted to refer specifically to *LoL* (see Appendix). For example, the phrase “…*as a result of the game*” was changed to “…*as a result of playing LoL*.” The scale demonstrated good internal consistency in a previous Latin American validation (*α* = .84–.85; *ω* = .82–.84) (23), and similar reliability indices were observed in the present study across all countries (*α* = .84–.86; *ω* = .84–.86).

### 2.3 Ethics

Prior to the survey, participants provided informed consent. They were assured that participation was voluntary, confidential, and anonymous; that they could withdraw at any time, and that the data would be used solely for research purposes.

The study adhered to ethical guidelines for research involving human participants. All participants provided voluntary informed consent, and the study ensured data confidentiality. It also complied with the ethical standards of the Declaration of Helsinki (41) and the American Psychological Association (APA) (42). The Ethics Committee of the Research Directorate at the University of Ibagué approved the study questionnaire under Act No. 14.

### 2.4 Procedure

In the present cross-sectional study, participants were recruited through non-probabilistic sampling via online platforms (*Discord, Reddit*, official gaming forums, *Instagram*, and *Facebook*). Inclusion criteria required participants to be *LoL* players aged ≥18 years and nationals of a Latin American country. Individuals who had played *LoL* for less than six months were excluded. Data were collected online via the *KoboToolbox* platform (43) between August 2022 and March 2023. Participants received no financial or non-financial compensation for taking part in the study.

### 2.5 Statistical analysis

Analyses were conducted using R version 4.4.3 (44) via RStudio (v2023.06.0) (45), using the packages “lavaan” for confirmatory factor analysis (CFA) (46), “semTools” for invariance testing (47), and “vcd” for categorical differences (48).

Differences in IGD prevalence by sex within each country were evaluated using chi-square (*χ*²) tests. To address small/unequal cells, a Monte Carlo (10,000) resampling under the null (hypergeometric, fixed margins) set *p* as the proportion with *χ*² ≥ observed (49, 50).

To infer the prevalence of IGD in the general population (πê_general_) from the observed gamer prevalence (πê_gamers_), the weighted-prevalence estimator was applied. The authors assumed 51.38% as the gamer population across Latin America based on available evidence (see 2).


$$\hat{\pi }general=0.5138\cdot \hat{\pi }gamers$$

Assuming negligible IGD among non-gamers, the variance of this estimator was then derived via the delta method (51):


$$Var(\hat{\pi }general)\approx {\left(0.5138\right)}^{2}\cdot Var(\hat{\pi }gamers),$$

where (52):


$$Var(\hat{\pi }gamers)=[\hat{\pi }gamers\;\cdot \;(1-\hat{\pi }gamers)]/ngamers$$

and 95 % confidence intervals were calculated as


$$\hat{\pi }general\pm 1.96\cdot \surd Var(\hat{\pi }general).$$

Percentage differences were quantified between probable IGD and non-probable IGD cases using a rigorous two-step approach. First, 2 × k contingency tables for each categorical variable using Pearson’s chi-square tests were analyzed. When expected cell counts were below five in 2 × 2 tables, Fisher’s exact test was applied. Effect sizes were reported as Cramer’s *V* (53).

Second, level-wise comparisons were conducted via two-sample proportion tests using the Agresti–Coull method to calculate 95% confidence intervals for absolute differences in proportions (52, 54). Moreover, *p-*values were adjusted for multiple comparisons using the Benjamini–Hochberg false discovery rate procedure (55). Descriptive statistics (mean, SD, skewness [g1], kurtosis [g2]) were calculated by country and assessed normality using g1 < ± 2 and g2 < ± 7 (56).

For the probable-IGD subgroup, item-level descriptive statistics included the number of valid responses, mean, standard deviation, median, interquartile range, observed range, uncorrected moment skewness, excess kurtosis, and response frequencies for each of the five response categories. Pearson inter-item correlations were calculated as the primary association estimates, and polychoric correlations were estimated as a complementary ordinal analysis. All nine items were complete; therefore, all calculations used the full probable-IGD subgroup (n = 1,248).

Across the probable IGD sample, confirmatory factor analyses (CFAs) were conducted to evaluate the internal structural validity of the IGDS9-SF across Latin American countries, applying robust maximum likelihood estimation (MLR). Model fit was assessed with multiple indices: Root Mean Square Error of Approximation (RMSEA), Standardized Root Mean Square Residual (SRMR), Comparative Fit Index (CFI), and Tucker-Lewis Index (TLI). Additionally, modification indices (MIs) were examined to identify any model-specific misspecifications and reported the chi-square statistic (*χ*²) to test overall model fit.

Measurement invariance was evaluated sequentially at the configural, metric, scalar, and strict levels. Consecutive models were compared using Satorra–Bentler scaled difference tests and changes in robust CFI and RMSEA. Invariance was considered supported when the decrease in CFI did not exceed.010 (ΔCFI ≥ −.010) and the increase in RMSEA did not exceed.015 (ΔRMSEA ≤.015) between consecutive models (57). Absolute fit indices were also considered jointly. When complete invariance was not supported, exploratory partial-invariance models were examined using robust score tests and standardized expected parameter changes. A constraint was eligible for release when the score test was significant (p <.05), the maximum absolute standardized expected parameter change was at least.10, and releasing the parameter significantly improved fit according to the robust nested-model comparison. At least four indicators remained invariant.

The residuals of Items 2 (withdrawal) and 3 (tolerance) were allowed to covary because both represent closely related dependence-like processes involving escalating gaming involvement and distress when gaming is reduced, potentially producing local dependence beyond the general IGD factor (58, 59).

The reliability of the IGDS9-SF was assessed using Cronbach’s alpha and McDonald’s omega coefficients. Multigroup confirmatory factor analyses (MGCFAs) were conducted to test measurement invariance across countries, sex, game server, and preferred game mode. Composite scores were compared descriptively across countries using one-way analysis of variance, with partial η² reported as the effect-size measure.

As a sensitivity analysis, the three prespecified one-factor models were re-estimated in the full sample of 10,151 participants, both overall and separately by country. Model 1 included all nine IGDS9-SF items; Model 2 added the residual covariance between Items 2 and 3; and Model 3 excluded Item 8 while retaining this residual covariance. Models 1 and 2 were compared using a Satorra–Bentler scaled difference test. Because Model 3 contained a different set of observed variables, its fit was compared descriptively and no likelihood-ratio, AIC, or BIC comparison with the nine-item models was interpreted. Measurement invariance of Model 3 was subsequently tested across country, sex, server, and preferred game mode using the same sequential criteria as in the primary analyses. The full-sample results were compared descriptively with those from the probable-IGD subgroup to assess the robustness of the findings to score-range restriction.

## 3 Results

### 3.1 Cross-country prevalence

**Table 1** presents IGD prevalence among gamers by country and sex. A significant sex difference was observed in Chile, with a higher prevalence among males (10.5%) than females (6.6%) [*χ*²(1) = 4.34, *p* = .040]. In contrast, in the remaining countries, prevalence was slightly higher among females, but none of these differences reached statistical significance [Mexico: *χ*²(1) = 1.06, *p* = .32; 12.8% females vs. 11.7% males; Colombia: *χ*²(1) = 0.10, *p* = .79; 14.7% females vs. 14.0% males; Peru: *χ*²(1) = 0.05, *p* = .90; 12.2% females vs. 11.7% males; Ecuador: *χ*²(1) = 0.66, *p* = .48; 18.6% females vs. 15.5% males]. In the combined sample, there was no sex difference: *χ²*(1) = 0.02, *p* = .89. IGD prevalence differed across countries, *χ²*(4) = 24.97, *p* <.001. The highest IGD prevalence was in Ecuador (16.1%), followed by Colombia (14.1%), Mexico (11.9%), Peru (11.8%), and Chile (9.5%).

**Table 1:** Prevalence in gamer population of internet gaming disorder by country and sex.

Country	Sex	n	Positive cases	Gamer prevalence (%)	χ² between sex (*p*-value)	General prevalence (%) [CI 95 %]
México	Total	5095	606	11.9 [11.0, 12.8]	1.06 (.32)	6.1 [5.7, 6.6]
	Male	4041	471	11.7 [10.7, 12.7]		6.0 [5.5, 6.5]
	Female	1054	135	12.8 [10.9, 15.0]		6.6 [5.5, 7.6]
Colombia	Total	2120	299	14.1 [12.7, 15.7]	0.10 (.79)	7.2 [6.5, 8.0]
	Male	1772	248	14 [12.4, 15.7]		7.2 [6.4, 8.0]
	Female	348	51	14.7 [11.2, 18.9]		7.5 [5.6, 9.4]
Perú	Total	1037	122	11.8 [9.9, 13.9]	0.05 (.90)	6.1 [5.1, 7.1]
	Male	849	99	11.7 [9.6, 14.1]		6.0 [4.9, 7.1]
	Female	188	23	12.2 [8.1, 18.0]		6.3 [3.9, 8.7]
Chile	Total	1276	121	9.5 [8.0, 11.3]	4.34 (.04)	4.9 [4.1, 5.7]
	Male	943	99	10.5 [8.7, 12.7]		5.4 [4.4, 6.4]
	Female	333	22	6.6 [4.3, 10.0]		3.4 [2.0, 4.8]
Ecuador	Total	623	100	16.1 [13.3, 19.2]	0.66 (.48)	8.3 [6.8, 9.8]
	Male	510	79	15.5 [12.5, 19.0]		7.9 [6.4, 9.6]
	Female	113	21	18.6 [12.1, 27.2]		9.6 [5.9, 13.2]
Total	Total	10151	1248	12.3 [11.7, 13.0]	0.02 (.89)	6.3 [6.0, 6.6]
	Male	8115	996	12.3 [11.6, 13.0]		6.3 [6.0, 6.7]
	Female	2036	252	12.4 [11.0, 13.9]		6.4 [5.6, 7.1]

CI confidence interval; *p < .05.

To estimate IGD prevalence in the general population, the gamer population (51.38%) was multiplied by each country’s IGD rate among gamers and applied the delta method to derive variances and 95% confidence intervals. This yielded estimated IGD prevalence rates of 6.11% (95% CI 5.66–6.57) in Mexico, 7.24% (95% CI 6.48–8.01) in Colombia, 6.06% (95% CI 5.05–7.07) in Peru, 4.88% (95% CI 4.05–5.71) in Chile, 8.27% (95% CI 6.79–9.76) in Ecuador, and 6.32% (95% CI 5.99–6.65) across Latin America (**Table 1**).

### 3.2 Characteristics of the probable IGD sample

As reported in the supplementary material (**Supplementary Table 1**), probable-IGD participants from Chile (*M* = 21.7 years, *SD* = 3.5) and México (*M* = 20.4 years, *SD* = 2.9) were the oldest. Males predominated in all countries (>70%). Most participants reported a high-school education. Regarding server use, players from Ecuador, Mexico, and Colombia primarily connected to LAN (Latin America North; 98–99%). Chile mostly used LAS (Latin America South; 97.5%), and Peruvian players were more evenly distributed (LAN 56.6%, LAS 43.3%).

**Table 2** compares the probable IGD group (*n* = 1,248) vs. non-probable IGD group (*n* = 8,903), and three variables differed: (i) preferred game mode, *χ²*(2) = 28.03, *p* <.001, *V* = .053. Probable IGD gamers favored ranked play (Mode 2 = 52.6% vs 44.9%; Δ = +7.7 pp, 95% CI 5.1–10.3; OR = 1.37 [1.21, 1.54]) and were less likely to play casual (25.3% vs 30.9%; Δ = −5.6 pp; OR = 0.76 [0.66, 0.87]); (ii) server preference, *χ²*(1) = 7.07, *p* = .008, *V* = .026. The probable-IGD group was less likely to use LAS than the non-probable-IGD group (14.3% vs. 17.3%; Δ = −3.0 pp; OR = 0.80 [0.67, 0.94]); (iii) educational level showed a small but significant imbalance, *χ²*(5) = 12.50, *p* = .030, *V* = .035. Probable IGD were overrepresented among bachelor’s degree holders (51.8% vs 47.1%; Δ = +4.7 pp; OR = 1.21 [1.07, 1.36]) and underrepresented at postgraduate level (12.5% vs 13.7%; Δ = −1.7 pp; OR = 0.86 [0.72, 1.04]). Sex did not differ, *χ²*(1) = 0.02, *p* = .90, *V* <.01 (OR = 0.99 [0.85, 1.15]).

**Table 2:** Sociodemographic and gaming characteristics of probable IGD and non-probable IGD participants.

Variable	Probable IGD n (%) | *M* (*SD*)	Non-probable IGD n (%) | *M* (*SD*)	Statistic | Δ	OR [IC 95%]
Total	1248 (100%)	8903 (100%)		
Age	20.3 ± 2.9	21 ± 3.2	-0.7	–
Sex			χ² = .02	
Male	996 (79.8%)	7119 (80%)	-0.2%	0.99 [0.85, 1.15]
Female	252 (20.2%)	1784 (20%)	0.2%	1.01 [0.87, 1.17]
Education level			χ² = 12.50*	
None	3 (0.2 %)	30 (0.3%)	-0.1%	0.71 [0.14, 2.30]
Primary basic	1 (0.1 %)	24 (0.3%)	-0.2%	1.82 [0.29, 11.82]
Basic high school	153 (12.3 %)	1098 (12.3%)	-0.1%	0.99 [0.82, 1.19]
Bachelor	647 (51.8 %)	4192 (47.1%)	4.8%*	1.21 [1.07, 1.36]
Undergraduate	292 (23.4 %)	2327 (26.1%)	-2.7%*	0.86 [0.75, 0.99]
Graduate	152 (12.5 %)	1232 (13.7%)	-1.7%	0.86 [0.72, 1.04]
Server			χ² = 7.07*	
LAN	1070 (85.7%)	7365 (82.7%)	3%*	1.26 [1.06, 1.49]
LAS	178 (14.3%)	1538 (17.3%)	-3%*	0.80 [0.67, 0.94]
Preferred game mode			χ² = 28.02*	
Mode 1	316 (25.3%)	2747 (30.9%)	-5.5%*	0.76 [0.66, 0.87]
Mode 2	657 (52.6%)	3992 (44.9%)	7.8%*	1.37 [1.21, 1.54]
Mode 3	275 (22%)	2164 (24.2%)	-2.3%	0.88 [0.76, 1.02]

Mode 1 = Normal Game in Summoner’s Rift, Flexible Qualifier; Mode 2 = Individual or duo qualification; Mode 3 = ARAM, URF/ARURF, Other rotary mode; *p < .05.

### 3.3 Item-level descriptive statistics and inter-item correlations of the IGDS9-SF by country

For the total sample (probable + non-probable IGD), **Supplementary Table 2** shows that Item 1 (preoccupation) had the highest mean across countries, suggesting many occasionally viewed the game as excessively time-consuming. Item 8 (coping) had the second-highest mean, reflecting a coping function. Item 9 (negative consequences) had the lowest mean and was rarely endorsed. Inter-item correlations were generally moderate. All items showed acceptable skew and kurtosis (*g1* < ± 2; *g2* < ± 7), meeting Finney and DiStefano’s (56) criteria. **Supplementary Table 2** also reports reliability by country (Cronbach’s *α*, McDonald’s *ω*).

Item-level descriptive statistics for the probable-IGD subgroup are reported in **Supplementary Table 3**. Item 1 showed the highest mean (M = 4.57, SD = 0.65), followed by Item 8 (M = 4.39, SD = 0.95), whereas Item 9 showed the lowest mean (M = 3.46, SD = 1.56). Overall item distributions remained within the prespecified skewness and kurtosis thresholds. Pearson inter-item correlations ranged from −.133 to.467, and the complementary polychoric correlations ranged from −.131 to.536 (**Supplementary Table 3D**). The generally weak inter-item associations are consistent with the restricted score variability produced by selecting participants who scored at or above the same IGDS9-SF threshold.

### 3.4 Validity bases on the internal structure of the IGDS9-SF in probable IGD participants

**Table 3** shows that the original one-factor model (Model 1) did not achieve acceptable fit in any of the five countries in the probable-IGD sample. Modification indices (60) were inspected and, on theoretical grounds, the residuals of Items 2 (withdrawal) and 3 (tolerance) were allowed to covary (Model 2); however, this model continued to show poor fit across the country samples. Model 3 was then tested by removing Item 8, based on conceptual/interpretive concerns reported in a previous Latin American general population sample (23). Fit improved markedly and was acceptable in most countries; Ecuador remained unacceptable (CFI = .83, TLI = .76), suggesting cultural/contextual differences in the latent structure. Model 3 was retained for subsequent psychometric analyses.

**Table 3:** Fit indices of IGDS9-SF measurement models among participants with probable IGD across Latin American countries.

Model and Country	Fit indices						
	χ^2^	df	*p*	CFI	TLI	SRMR	RMSEA
Model 1							
México (n = 606)	57.007	27	<.001	.89	.86	.040	.043
Colombia (n = 299)	63.204	27	<.001	.81	.74	.054	.067
Perú (n = 122)	40.792	27	.043	.79	.72	.067	.065
Chile (n = 121)	45.516	27	.014	.77	.70	.076	.075
Ecuador (n = 100)	36.518	27	.104	.80	.73	.077	.059
Model 2							
México (n = 606)	50.032	26	.003	.91	.88	.038	.039
Colombia (n = 299)	33.531	26	.156	.96	.98	.041	.030
Perú (n = 122)	41.900	26	.025	.80	.72	.066	.071
Chile (n = 121)	30.258	26	.163	.93	.91	.070	.047
Ecuador (n = 100)	37.429	26	.068	.77	.69	.078	.066
Model 3							
México (n = 606)	34.544	19	.026	.95	.93	.033	.035
Colombia (n = 299)	24.315	19	.184	.97	.96	.039	.031
Perú (n = 122)	27.339	19	.097	.92	.89	.058	.060
Chile (n = 121)	26.991	19	.105	.94	.91	.065	.059
Ecuador (n = 100)	29.693	19	.056	.83	.76	.074	.075

χ^2^, Chi square; df, degrees of freedom; SRMR, Standardized Root Mean Square Residual; TLI, Tucker-Lewis Index; CFI, Comparative Fit Index; RMSEA, Root Mean Square Error of Approximation; BIC, Sample-size adjusted Bayesian; ω, Omega de McDonald. Model 1 = Unidimensional model; Model 2 = Unidimensional model with one correlated error 2~3; Model 3 = Unidimensional model without item 8 with one correlated error 2~3.

### 3.5 Measurement invariance according to country, sex, server, and game mode among probable IGD participants

At the country level, metric invariance met the prespecified change criteria (ΔCFI = .012; ΔRMSEA = −.009), but scalar invariance was not supported (ΔCFI = −.034; ΔRMSEA = .005). Adding equality constraints on residual variances further reduced CFI from.910 to.860 (ΔCFI = −.050) and increased RMSEA from.039 to.045 (ΔRMSEA = .006). The strict model also showed inadequate absolute fit according to CFI (.860) and TLI (.893). Moreover, the retained model showed poor country-specific fit in Ecuador (CFI = .83; TLI = .76). Exploratory partial-invariance analyses were conducted after scalar invariance failed. Releasing candidate intercept constraints identified through robust score tests and standardized expected parameter changes did not yield a defensible partial scalar invariance model under the prespecified criteria. Therefore, only metric invariance across countries was retained. Partial strict invariance was not interpreted, and equivalence of item intercepts, residual variances, or mean scores across the five countries was not assumed. Strict invariance was supported across sex and server groups according to the corrected consecutive change indices reported in **Table 4**. For preferred game mode, metric invariance was supported, but adding equality constraints on intercepts produced a substantial decline in fit (ΔCFI = −.035; ΔRMSEA = .006). Consequently, scalar and strict invariance across preferred game modes were not established, and comparisons requiring equal item intercepts across these groups were not interpreted.

**Table 4:** Scale measurement invariance models of the IGDS9-SF among participants with probable IGD.

Invariance models	χ^2^	df	*p*	TLI	CFI	SRMR	RMSEA	Δχ^2^	Δdf	ΔCFI	ΔRMSEA
By country											
Configural	141.391	95	.001	.900	.932	.044	.043	–	–	–	–
Metric	158.328	123	.017	.936	.944	.049	.034	22.259	28	.012	-.009
Scalar	208.242	151	.001	.916	.910	.056	.039	50.146	28	-.034	.005
Strict	268.067	183	.000	.893	.860	.073	.045	57.253	32	-.050	.005
By sex											
Male	69.207	19	.000	.884	.921	.034	.045	–	–	–	
Female	22.619	19	.225	.968	.978	.033	.025	–	–	–	–
Configural	95.393	38	.000	.878	.917	.037	.048	–	–	–	–
Metric	92.507	45	.000	.909	.927	.038	.042	2.478	7	.009	-.006
Scalar	104.231	52	.000	.914	.920	.040	.040	11.606	7	-.007	-.001
Strict	105.552	60	.000	.928	.932	.041	.036	4.134	8	.012	-.005
According to server											
LAN	64.358	19	.000	.892	.927	.034	.043	–	–	–	–
LAS	25.598	19	.142	.940	.959	.039	.035	–	–	–	–
Configural	76.189	38	.000	.917	.944	.034	.039	–	–	–	–
Metric	74.745	45	.004	.942	.953	.034	.033	2.357	7	.010	-.006
Scalar	84.831	52	.003	.945	.949	.036	.032	10.006	7	-.004	-.001
Strict	94.467	60	.003	.948	.944	.040	.031	10.342	8	-.005	-.001
According to game mode											
Mode 1	20.882	19	.343	.984	.989	.032	.016	–	–	–	–
Mode 2	44.697	19	.001	.904	.935	.035	.041	–	–	–	–
Mode 3	28.782	19	.069	.937	.957	.040	.039	–	–	–	–
Configural	91.406	57	.003	.921	.946	.038	.038	–	–	–	–
Metric	103.371	71	.007	.939	.949	.043	.034	12.651	14	.002	-.005
Scalar	140.284	85	.000	.914	.913	.049	.040	37.905	14	-.035	.006
Strict	158.913	101	.000	.921	.905	.057	.038	20.143	16	-.008	-.002

CFI, Comparative Fit Index; TLI, Tucker-Lewis Index; RMSEA, Root Mean Square Error of Approximation; SRMR, Standardized Root Mean Square Residual. ΔCFI and ΔRMSEA represent changes relative to the immediately preceding invariance model. Invariance was considered supported when ΔCFI was greater than or equal to −.010 and ΔRMSEA was less than or equal to.015.

### 3.6 Sensitivity analyses in the full sample

Selection of participants using the IGDS9-SF cut-off substantially restricted score variability. The standard deviation of the total score was 3.74 in the probable-IGD subgroup compared with 7.77 in the full sample, and the mean Pearson inter-item correlation was.049 compared with.382, respectively.

In the full sample, the original nine-item model showed mixed fit, robust χ²(27) = 2234.04, p <.001, CFI = .915, TLI = .887, RMSEA = .099, 90% CI [.095,.102], SRMR = .050. Allowing the residuals of Items 2 and 3 to covary significantly improved fit, robust Δχ²(1) = 531.34, p <.001; Model 2: CFI = .940, TLI = .917, RMSEA = .085, 90% CI [.082,.089], SRMR = .043. Model 3, which excluded Item 8 while retaining this residual covariance, yielded CFI = .951, TLI = .927, RMSEA = .086, 90% CI [.082,.091], and SRMR = .041. Relative to Model 2, excluding Item 8 improved CFI by.011 and SRMR by.002 but slightly increased RMSEA by.001. Therefore, the advantage of removing Item 8 was smaller and depended on the fit index considered in the full sample (**Supplementary Table 4A**).

Using Model 3, the consecutive ΔCFI and ΔRMSEA criteria supported strict invariance across country, sex, server, and preferred game mode in the full sample. Nevertheless, configural RMSEA values were approximately.086 across these analyses, indicating mixed absolute fit despite acceptable CFI, TLI, and SRMR values. These sensitivity results show that the Item 2 and Item 3 residual dependence was reproduced in the full sample, whereas the country and game-mode invariance problems observed in the probable-IGD subgroup were substantially attenuated (**Supplementary Table 4B**).

**Figure 1:**
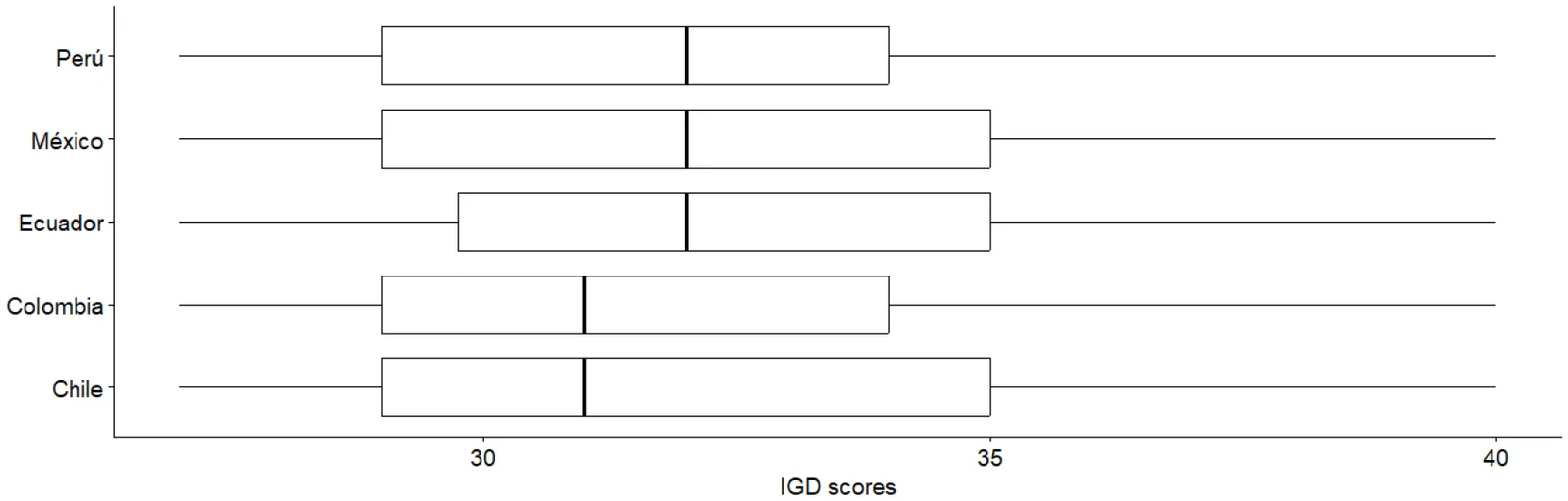
Distribution of internet gaming disorder scores by country among participants with probable IGD. IGD, Internet gaming disorder. No statistically significant differences were found between countries (p >.05).

**Figure 1** illustrates the distribution of IGDS9-SF scores among participants with probable IGD across countries. Although participants from Colombia and Chile showed slightly lower IGD scores, these differences were small and statistically non-significant (*F*(4, 1243) = 0.15, *p* = .965, partial η² = .00, 90% CI [.00, 1.00]).

## 4 Discussion

The present study provides the first multinational estimate of IGD prevalence in Latin America and the first test of the IGDS9-SF’s cross-national measurement invariance in a high-risk gaming sample. Metric invariance was supported across the five countries, and strict invariance was supported across sex and server groups. Prior invariance research on the scale has been conducted exclusively in low-risk samples (13, 22–27); the present findings extend evidence of its consistent cross-national functioning to a high-symptom population for the first time.

The observed rate of probable IGD among recruited players was 12.3%, and the exploratory general-population extrapolation was 6.3%, with country-specific rates from 4.9% (Chile) to 8.3% (Ecuador). This finding narrows the wide prevalence range previously reported in Latin America (1.1%–38.2%) (17). The only reliable comparative data available concerns IGD prevalence in Mexican university students, reported at 5.2% (61), which aligns with the current study’s estimate for Mexico (6.1%). Moreover, overall prevalence estimate for Latin America aligns with global DSM-5-based figures (~6.7%) (15) but exceeds those from ICD-11 on general-population studies (~3.3%) (62), reinforcing the need for diagnostic consensus to ensure comparability (63). The DSM-5 prevalence likely reflects its lower diagnostic threshold and inclusion of behavioral indicators that overlap with normative gaming engagement. Intraregional disparities may reflect socioeconomic factors. However, the higher rate observed in Ecuador should be interpreted cautiously due to methodological limitations (e.g., small sample size).

Unlike prior findings identifying younger age and male sex as risk factors (64, 65), the present study found no significant differences in these demographics between probable IGD and non-probable IGD players. Therefore, additional explanations should be considered, such as the growing participation of females in competitive gaming and potential shifts in gender-related gaming motivations and coping styles. Instead, the main disparities were related to education, server, and preferred game mode. Notably, bachelor degree students showed a higher prevalence of probable IGD, possibly because transitional academic stages intensify the use of gaming as a coping mechanism for stress or social demands (66, 67).

Regionally, a higher likelihood of probable IGD was found among players on the LAN server compared to LAS. Because servers use closely mirrored country of residence, this pattern likely reflects broader cross-country differences rather than server choice itself. One possible explanation, consistent with compensatory internet use theory (33), is that cross-country structural stressors and socioeconomic disparities may increase reliance on gaming as a coping outlet (68–71); however, this interpretation remains speculative given the server-country overlap. Competitive game mode was also strongly associated with probable IGD, supporting the association between competitive environments and problematic use (72). Overall, these patterns highlight the relevance of contextual and behavioral factors over demographics in explaining IGD vulnerability.

Applying the IGDS9-SF within a standardized behavioral context (i.e., *LoL* players) helps reduce measurement error and allows for more precise cross-national comparisons by minimizing genre-specific confounds. Across countries, items reflecting more common experiences (e.g., preoccupation, playing to escape negative moods) were endorsed more frequently, whereas items capturing serious external consequences (e.g., damaged relationships, jeopardized opportunities) were less often affirmed. This consistent hierarchy supports measurement invariance by showing that participants across cultural contexts conceptualize and respond to the scale in highly similar ways.

Crucially, the present study is the first to test the measurement invariance of the Latin American version of the IGDS9-SF within a high-risk (probable IGD) sample. Metric invariance was supported across countries, confirming comparable factor structure and loadings, but scalar and strict invariance were not, precluding direct comparison of observed severity scores between countries. Strict invariance was supported across sex and server, permitting more direct comparisons for these variables. These results are consistent with recent regional evidence for the scale’s unidimensional structure and measurement invariance across demographic groups, particularly when Item 8 is excluded (23). Overall, the model demonstrates sufficient stability to support valid cross-national comparisons of the underlying IGD construct at the metric level; the results for Ecuador warrant caution given its smaller sample size, comparatively weaker model fit (**Table 3**), and potential sampling biases.

To test whether these invariance limitations reflected the restricted score variability of a high-risk subgroup rather than a true scale property, sensitivity analyses were conducted in the full sample (N = 10,151). Strict invariance was supported across country, sex, server, and game mode, though configural RMSEA remained around.086, indicating mixed absolute fit. This suggests that invariance is harder to establish specifically among high-symptom players, the population most relevant to clinical screening, and that this difficulty likely reflects restricted score variance rather than a genuine lack of cross-national equivalence.

The analysis identified Item 8 (escapism) as a psychometric weakness. While a prior analysis using the same broader data collection identified this pattern in the general population (23), the present study is the first to test it specifically within a high-symptom, high-risk sample, where diagnostic precision is most critical (21, 31, 32). Although highly endorsed, this item has shown poor specificity and low clinical utility in previous studies (13, 30). Accordingly, its removal significantly improved the model’s fit and internal consistency.

This suggests that escapism may function more as a general gaming motivation than as a core pathological symptom (20, 26). In high-stress toxic contexts such as competitive MOBAs, endorsing Item 8 may capture situational coping responses rather than a stable pathology (34, 73). However, improved model fit following the removal of Item 8 is psychometric evidence and does not, by itself, establish that escapism is adaptive or that it should be excluded as a diagnostic criterion (74, 75); clarifying its clinical status would require additional longitudinal and diagnostic-accuracy evidence. Future diagnostic frameworks should nonetheless consider differentiating adaptive from maladaptive forms of escapism, informed by such evidence.

A correlated error was also identified between Item 2 (withdrawal) and Item 3 (tolerance), a symptom pattern distinct from that of non-problematic players (23). This suggests that in high-risk populations, these two criteria function as a reinforcing cycle: players may increase their gaming time (tolerance) to alleviate the negative emotions experienced when trying to stop or reduce play (withdrawal) (58, 59). The measurement invariance findings of the IGDS9-SF in Latin America have significant research and clinical implications. For researchers, it provides a valid tool for comparing IGD severity across countries within high-risk populations at the metric level, supporting accurate cross-national prevalence estimates and the study of sociocultural influences. For clinicians and policymakers, it serves as a reliable screener to help address the recognition and treatment gap for IGD in the region, where research and clinical infrastructure for behavioral addictions remain limited (76). Integrating this instrument into clinical practice, coupled with culturally sensitive education and a policy focus on prevention, could enable timely, evidence-based care and reduce the disorder’s long-term burden.

The present study has several limitations. First, regarding design and measurement, the cross-sectional approach precludes causal inferences, and reliance on self-reports may introduce recall and social desirability biases. A key methodological limitation is that the prevalence estimate was based on the full nine-item scale, which included Item 8, an item the analyses identified as psychometrically weak. Additionally, defining the probable-IGD subgroup via the same IGDS9-SF cut-off later analyzed psychometrically restricted score variability, likely contributing to the country- and game-mode-level invariance limitations. Moreover, the absence of formal clinical diagnoses limits the definitive classification of cases. Finally, regarding sampling and generalizability, the non-probabilistic sample of LoL players limits generalizability to other gaming contexts, and the reported general-population prevalence should accordingly be interpreted as an exploratory extrapolation rather than a representative epidemiological estimate. The low proportion of female participants (19.9%) and unequal country sample sizes further limit representativeness.

Future research should address several key areas. First, studies should continue to focus on underexplored IGD high-risk populations and extend invariance testing to other Latin American countries. Second, dedicated research is needed to understand the distinctive symptom pattern found in Ecuador. Third, any modification to the scale, such as removing Item 8, requires substantial further validation, including establishing new cut-off scores and testing across diverse gamer populations (not only *LoL* players). Finally, new IGD instruments should be developed with qualitative input from clinically diagnosed individuals and validated using rigorous methodologies, such as item response theory.

## 5 Conclusion

The present study provides the first multinational estimates of IGD prevalence among *LoL* gamers in Latin America (12.3%, with an exploratory general-population extrapolation of 6.3%) and provides the first evidence of the IGDS9-SF’s measurement invariance in a high-risk regional sample, supporting its use for comparing IGD severity within high-risk groups across countries at the metric level, and more directly across sex and server. Its most critical contribution is the evidence that a core DSM-5-TR diagnostic criterion—escapism (Item 8)—is psychometrically weak, even among high-symptom gamers. These results underscore the need for further research to re-examine the DSM-5 framework for IGD. Systematic screening and culturally sensitive prevention strategies remain essential to address the under-recognition of IGD in the region.

## Data Availability

The datasets presented in this study can be found in online repositories. The names of the repository/repositories and accession number(s) can be found below: https://osf.io/mvxtz/overview?view_only=8a4c4017ea694cfab4aa4ac9de4f0eb4.

## Appendix

**Table:** Spanish Linguistically Adapted Version of IGDS9-SF.

Los siguientes enunciados hacen referencia a tu actividad con League of Legends durante el último año.					
1. Creo que jugar League of Legends es una de las actividades que más consume tiempo en mi vida	1	2	3	4	5
2. Cuando trato de bajar el ritmo de juego o suspenderlo por un tiempo, me siento irritado, nervioso o triste	1	2	3	4	5
3. Siento la necesidad de pasar cada vez más tiempo en el juego para sentirme bien o satisfecho	1	2	3	4	5
4. A menudo me propongo jugar menos pero acabo por no lograrlo	1	2	3	4	5
5. He perdido interés en otras aficiones o pasatiempos debido a jugar LoL	1	2	3	4	5
6. He continuado jugando a pesar de los problemas que me genera con otras personas	1	2	3	4	5
7. He ocultado o mentido a mi familia, terapeutas u otras personas sobre la cantidad de tiempo que dedico al juego	1	2	3	4	5
* 8. Juego para escapar temporalmente o aliviar un estado de ánimo negativo (por ejemplo, culpa, miedo, frustración).	1	2	3	4	5
9. He puesto en riesgo de perder, o he perdido una relación importante, un trabajo o una oportunidad educativa o profesional por jugar LoL	1	2	3	4	5

* Item was eliminated in the measurement invariance analysis.

1 = Nunca; 2 = Rara vez; 3 = Ocasionalmente; 4 = Frecuentemente; 5 = Muy frecuentemente
