# Supplementary material for: Prevalence of internet gaming disorder and measurement invariance of the IGDS9-SF in a high-risk Latin American sample
Source: Front Psychiatry. 2026 Sep 10;17:1913038. doi: 10.3389/fpsyt.2026.1913038 (PMC13603059; doi:10.3389/fpsyt.2026.1913038)
Supplement: Supplementary file 1 [file DataSheet1.pdf]

# Supplementary Material

## 1 Supplementary Data

**Table S1**

*Sociodemographic and Gaming Characteristics of Participants with Probable IGD by Country*

| | **México**<br>n (%) \| *M* (*SD*) | **Colombia**<br>n (%) \| *M* (*SD*) | **Perú**<br>n (%) \| *M* (*SD*) | **Chile**<br>n (%) \| *M* (*SD*) | **Ecuador**<br>n (%) \| *M* (*SD*) |
|---|---|---|---|---|---|
| **Total** | 606 (48.6%) | 299 (23.9%) | 122 (9.8%) | 121 (9.7%) | 100 (8.0%) |
| **Age** | 20.4 ± 2.9 | 19.8 ± 2.3 | 19.6 ± 2.6 | 21.7 ± 3.5 | 19.8 ± 2.2 |
| **Sex** | | | | | |
| Male | 471 (77%) | 248 (82.9%) | 99 (81.1%) | 99 (81.8%) | 79 (79%) |
| Female | 135 (22.3%) | 51 (17.1%) | 23 (18.9%) | 22 (18.2%) | 21 (21%) |
| **Education level** | | | | | |
| None | 2 (0.3%) | 1 (0.3%) | 0 (0%) | 0 (0%) | 0 (0%) |
| Primary basic | 0 (0%) | 0 (0%) | 0 (0%) | 1 (.8%) | 0 (0%) |
| Basic high school | 31 (5.1%) | 19 (6.4%) | 51 (41.8%) | 47 (38.8%) | 5 (5%) |
| Bachelor | 342 (56.4%) | 181 (60.5%) | 36 (29.5%) | 21 (17.4%) | 67 (67%) |
| Undergraduate | 126 (20.8%) | 85 (28.4%) | 29 (23.8%) | 34 (28.1%) | 18 (18%) |
| Graduate | 105 (17.3%) | 13 (4.3%) | 6 (4.9%) | 18 (14.8%) | 10 (10%) |
| **Server** | | | | | |
| LAN | 604 (99.7%) | 296 (99%) | 69 (56.6%) | 3 (2.5%) | 98 (98%) |
| LAS | 2 (0.3%) | 3 (1%) | 53 (43.4%) | 118 (97.5%) | 2 (2%) |
| **Preferred game mode** | | | | | |
| Mode 1 | 173 (28.5%) | 69 (23.1%) | 28 (23%) | 25 (20.7%) | 21 (21%) |
| Mode 2 | 298 (49.2%) | 170 (56.9%) | 68 (55.7%) | 67 (55.4%) | 54 (54%) |
| Mode 3 | 135 (22.3%) | 60 (20.1%) | 26 (21.3%) | 29 (24%) | 25 (25%) |

Mode 1 = Normal Game in Summoner's Rift, Flexible Qualifier; Mode 2 = Individual or duo
ication; Mode 3 = ARAM, URF/ARURF, Other rotary mode; * $p < .05$.

**Table S2**

*Item-Level Descriptive Statistics and Inter-Item Correlations of the IGDS9-SF by Country (n=10151)*

| Country (Reliability) | Item | (λ) | M | SD | g1 | g2 | IGD1 | IGD2 | IGD3 | IGD4 | IGD5 | IGD6 | IGD7 | IGD8 | IGD9 |
|---|---|---|---|---|---|---|---|---|---|---|---|---|---|---|---|
| México (α = .84; ω = .84) | IGD1 | .52 | 3.57 | 1.09 | -.26 | -.75 | 1 | .41 | .41 | .41 | .41 | .28 | .24 | .22 | .23 |
| | IGD2 | .79 | 2.10 | 1.18 | .91 | -.08 | | 1 | .71 | .62 | .51 | .30 | .34 | .30 | .33 |
| | IGD3 | .82 | 2.06 | 1.19 | .97 | -.02 | | | 1 | .65 | .55 | .31 | .35 | .33 | .34 |
| | IGD4 | .77 | 2.21 | 1.31 | .82 | -.51 | | | | 1 | .55 | .32 | .37 | .30 | .33 |
| | IGD5 | .69 | 2.32 | 1.41 | .66 | -.93 | | | | | 1 | .32 | .38 | .29 | .39 |
| | IGD6 | .44 | 2.77 | 1.47 | .19 | -1.36 | | | | | | 1 | .34 | .30 | .29 |
| | IGD7 | .50 | 1.77 | 1.23 | 1.47 | .91 | | | | | | | 1 | .35 | .45 |
| | IGD8 | .43 | 2.94 | 1.48 | .04 | -1.39 | | | | | | | | 1 | .31 |
| | IGD9 | .48 | 1.72 | 1.23 | 1.61 | 1.28 | | | | | | | | | 1 |
| Colombia (α = .84; ω = .84) | IGD1 | .53 | 3.61 | 1.06 | -.32 | -.66 | 1 | .42 | .40 | .43 | .39 | .27 | .25 | .26 | .25 |
| | IGD2 | .80 | 2.10 | 1.15 | .88 | -.06 | | 1 | .73 | .62 | .49 | .32 | .34 | .31 | .37 |
| | IGD3 | .82 | 2.11 | 1.17 | .89 | -.07 | | | 1 | .64 | .53 | .32 | .36 | .32 | .38 |
| | IGD4 | .78 | 2.27 | 1.31 | .71 | -.67 | | | | 1 | .59 | .32 | .35 | .30 | .36 |
| | IGD5 | .68 | 2.42 | 1.41 | .54 | -1.06 | | | | | 1 | .30 | .36 | .25 | .41 |
| | IGD6 | .44 | 3.00 | 1.44 | -.03 | -1.33 | | | | | | 1 | .36 | .31 | .29 |
| | IGD7 | .49 | 1.91 | 1.32 | 1.22 | .12 | | | | | | | 1 | .33 | .45 |
| | IGD8 | .42 | 3.04 | 1.47 | -.06 | -1.37 | | | | | | | | 1 | .32 |
| | IGD9 | .51 | 1.84 | 1.27 | 1.34 | .48 | | | | | | | | | 1 |
| Perú (α = .85; ω = .85) | IGD1 | .51 | 3.43 | .99 | -.13 | -.51 | 1 | .42 | .41 | .39 | .37 | .29 | .22 | .24 | .21 |
| | IGD2 | .81 | 2.21 | 1.12 | .72 | -.19 | | 1 | .72 | .64 | .53 | .38 | .39 | .31 | .34 |
| | IGD3 | .82 | 2.20 | 1.16 | .77 | -.23 | | | 1 | .63 | .56 | .37 | .36 | .35 | .36 |
| | IGD4 | .77 | 2.31 | 1.20 | .61 | .63 | | | | 1 | .58 | .38 | .36 | .28 | .33 |
| | IGD5 | .70 | 2.39 | 1.30 | .56 | -.84 | | | | | 1 | .31 | .44 | .29 | .39 |
| | IGD6 | .50 | 2.75 | 1.37 | .17 | -1.21 | | | | | | 1 | .35 | .39 | .28 |
| | IGD7 | .53 | 1.89 | 1.24 | 1.20 | .23 | | | | | | | 1 | .40 | .49 |
| | IGD8 | .44 | 3.00 | 1.40 | -.05 | -1.27 | | | | | | | | 1 | .31 |
| | IGD9 | .48 | 1.72 | 1.17 | 1.54 | 1.22 | | | | | | | | | 1 |
| Chile (α = .84; ω = .85) | IGD1 | .54 | 3.41 | 1.14 | -.14 | -.88 | 1 | .41 | .43 | .45 | .42 | .26 | .22 | .19 | .25 |
| | IGD2 | .77 | 1.87 | 1.11 | 1.19 | .53 | | 1 | .71 | .61 | .51 | .35 | .32 | .28 | .38 |
| | IGD3 | .80 | 1.91 | 1.15 | 1.21 | .57 | | | 1 | .63 | .57 | .33 | .33 | .29 | .39 |
| | IGD4 | .80 | 2.06 | 1.26 | 1.01 | -.08 | | | | 1 | .63 | .37 | .41 | .29 | .43 |
| | IGD5 | .73 | 2.28 | 1.41 | .73 | -.84 | | | | | 1 | .33 | .38 | .29 | .42 |
| | IGD6 | .47 | 2.60 | 1.47 | .36 | -1.27 | | | | | | 1 | .34 | .26 | .34 |
| | IGD7 | .50 | 1.57 | 1.06 | 1.96 | 2.93 | | | | | | | 1 | .33 | .46 |
| | IGD8 | .39 | 2.81 | 1.45 | .15 | -1.31 | | | | | | | | 1 | .29 |
| | IGD9 | .55 | 1.60 | 1.15 | 1.91 | 2.44 | | | | | | | | | 1 |
| Ecuador (α = .86; ω = .86) | IGD1 | .48 | 3.76 | 1.03 | -.36 | -.68 | 1 | .41 | .34 | .37 | .39 | .34 | .27 | .24 | .25 |
| | IGD2 | .81 | 2.21 | 1.21 | .79 | -.31 | | 1 | .71 | .66 | .53 | .37 | .45 | .37 | .36 |
| | IGD3 | .82 | 2.22 | 1.24 | .79 | -.39 | | | 1 | .69 | .55 | .40 | .40 | .38 | .35 |

| | | | | | | | | | | | | | | |
|---|---|---|---|---|---|---|---|---|---|---|---|---|---|---|
| IGD4 | .80 | 2.37 | 1.36 | .61 | -.88 | | | | 1 | .59 | .36 | .38 | .38 | .33 |
| IGD5 | .70 | 2.44 | 1.38 | .51 | -.99 | | | | | 1 | .39 | .42 | .29 | .39 |
| IGD6 | .53 | 2.88 | 1.48 | .10 | -1.39 | | | | | | 1 | .43 | .32 | .39 |
| IGD7 | .57 | 1.93 | 1.30 | 1.15 | .01 | | | | | | | 1 | .39 | .50 |
| IGD8 | .49 | 3.01 | 1.49 | -.03 | -1.39 | | | | | | | | 1 | .30 |
| IGD9 | .50 | 1.76 | 1.23 | 1.53 | 1.09 | | | | | | | | | 1 |

**Table S3**

*Item-Level Descriptive Statistics, Response Distributions, Inter-Item Correlations, and Reliability of the IGDS9-SF Among Participants With Probable IGD*

*Panel A. Overall Item-Level Descriptive Statistics*

| Item | n | M | SD | Mdn | IQR | Min–Max | $g_1$ | $g_2$ |
|---|---|---|---|---|---|---|---|---|
| Item 1 | 1,248 | 4.57 | 0.65 | 5.00 | 1.00 | 2–5 | -1.34 | 1.01 |
| Item 2 | 1,248 | 3.82 | 1.05 | 4.00 | 2.00 | 1–5 | -0.63 | -0.25 |
| Item 3 | 1,248 | 3.94 | 1.02 | 4.00 | 2.00 | 1–5 | -0.74 | -0.15 |
| Item 4 | 1,248 | 4.20 | 0.97 | 4.00 | 1.00 | 1–5 | -1.21 | 1.08 |
| Item 5 | 1,248 | 4.30 | 0.94 | 5.00 | 1.00 | 1–5 | -1.48 | 1.95 |
| Item 6 | 1,248 | 4.32 | 0.94 | 5.00 | 1.00 | 1–5 | -1.52 | 2.09 |
| Item 7 | 1,248 | 3.55 | 1.50 | 4.00 | 3.00 | 1–5 | -0.63 | -1.06 |
| Item 8 | 1,248 | 4.39 | 0.95 | 5.00 | 1.00 | 1–5 | -1.70 | 2.50 |
| Item 9 | 1,248 | 3.46 | 1.56 | 4.00 | 3.00 | 1–5 | -0.52 | -1.28 |

*Panel B. Overall Response Frequencies, n (%)*

| Item | n | 1 | 2 | 3 | 4 | 5 |
|---|---|---|---|---|---|---|
| Item 1 | 1,248 | 0 (0.0) | 6 (0.5) | 91 (7.3) | 335 (26.8) | 816 (65.4) |
| Item 2 | 1,248 | 33 (2.6) | 105 (8.4) | 303 (24.3) | 418 (33.5) | 389 (31.2) |
| Item 3 | 1,248 | 23 (1.8) | 98 (7.9) | 258 (20.7) | 419 (33.6) | 450 (36.1) |
| Item 4 | 1,248 | 25 (2.0) | 51 (4.1) | 180 (14.4) | 387 (31.0) | 605 (48.5) |
| Item 5 | 1,248 | 26 (2.1) | 43 (3.4) | 136 (10.9) | 369 (29.6) | 674 (54.0) |
| Item 6 | 1,248 | 26 (2.1) | 40 (3.2) | 135 (10.8) | 354 (28.4) | 693 (55.5) |
| Item 7 | 1,248 | 224 (17.9) | 99 (7.9) | 163 (13.1) | 286 (22.9) | 476 (38.1) |
| Item 8 | 1,248 | 28 (2.2) | 44 (3.5) | 119 (9.5) | 283 (22.7) | 774 (62.0) |
| Item 9 | 1,248 | 257 (20.6) | 113 (9.1) | 149 (11.9) | 253 (20.3) | 476 (38.1) |

**Table S3 (continued)**

*Panel C. Item-Level Descriptive Statistics by Country*

| Country | Item | n | M | SD | Mdn | IQR | Min–Max | $g_1$ | $g_2$ |
|---|---|---|---|---|---|---|---|---|---|
| Mexico | Item 1 | 606 | 4.58 | 0.64 | 5.00 | 1.00 | 2–5 | -1.30 | 0.64 |
| | Item 2 | 606 | 3.87 | 1.04 | 4.00 | 2.00 | 1–5 | -0.71 | -0.15 |
| | Item 3 | 606 | 3.95 | 0.98 | 4.00 | 2.00 | 1–5 | -0.70 | -0.17 |
| | Item 4 | 606 | 4.19 | 1.00 | 4.00 | 1.00 | 1–5 | -1.22 | 0.95 |
| | Item 5 | 606 | 4.30 | 0.95 | 5.00 | 1.00 | 1–5 | -1.48 | 1.93 |
| | Item 6 | 606 | 4.29 | 0.99 | 5.00 | 1.00 | 1–5 | -1.51 | 1.89 |
| | Item 7 | 606 | 3.56 | 1.52 | 4.00 | 3.00 | 1–5 | -0.64 | -1.08 |
| | Item 8 | 606 | 4.43 | 0.93 | 5.00 | 1.00 | 1–5 | -1.81 | 2.92 |
| | Item 9 | 606 | 3.46 | 1.59 | 4.00 | 3.00 | 1–5 | -0.51 | -1.33 |
| Colombia | Item 1 | 299 | 4.59 | 0.62 | 5.00 | 1.00 | 2–5 | -1.34 | 1.04 |
| | Item 2 | 299 | 3.72 | 1.05 | 4.00 | 2.00 | 1–5 | -0.58 | -0.20 |
| | Item 3 | 299 | 3.88 | 1.07 | 4.00 | 2.00 | 1–5 | -0.67 | -0.34 |
| | Item 4 | 299 | 4.17 | 0.97 | 4.00 | 1.00 | 1–5 | -1.13 | 0.86 |
| | Item 5 | 299 | 4.26 | 0.96 | 5.00 | 1.00 | 1–5 | -1.49 | 1.98 |
| | Item 6 | 299 | 4.36 | 0.84 | 5.00 | 1.00 | 1–5 | -1.45 | 2.18 |
| | Item 7 | 299 | 3.62 | 1.48 | 4.00 | 2.50 | 1–5 | -0.71 | -0.95 |
| | Item 8 | 299 | 4.35 | 0.98 | 5.00 | 1.00 | 1–5 | -1.58 | 1.86 |
| | Item 9 | 299 | 3.53 | 1.54 | 4.00 | 3.00 | 1–5 | -0.63 | -1.14 |
| Peru | Item 1 | 122 | 4.41 | 0.74 | 5.00 | 1.00 | 2–5 | -1.06 | 0.53 |
| | Item 2 | 122 | 3.93 | 0.92 | 4.00 | 2.00 | 1–5 | -0.44 | -0.47 |
| | Item 3 | 122 | 4.03 | 0.89 | 4.00 | 2.00 | 2–5 | -0.42 | -0.88 |
| | Item 4 | 122 | 4.10 | 0.92 | 4.00 | 1.00 | 1–5 | -1.02 | 0.92 |

| Country | Item | n | M | SD | Mdn | IQR | Min–Max | $g_1$ | $g_2$ |
|---|---|---|---|---|---|---|---|---|---|
| | Item 5 | 122 | 4.22 | 0.93 | 4.00 | 1.00 | 1–5 | -1.25 | 1.33 |
| | Item 6 | 122 | 4.19 | 1.04 | 4.50 | 1.00 | 1–5 | -1.40 | 1.62 |
| | Item 7 | 122 | 3.73 | 1.35 | 4.00 | 2.00 | 1–5 | -0.88 | -0.41 |
| | Item 8 | 122 | 4.34 | 0.91 | 5.00 | 1.00 | 1–5 | -1.69 | 3.03 |
| | Item 9 | 122 | 3.39 | 1.43 | 4.00 | 2.00 | 1–5 | -0.46 | -1.05 |
| Chile | Item 1 | 121 | 4.58 | 0.67 | 5.00 | 1.00 | 2–5 | -1.64 | 2.57 |
| | Item 2 | 121 | 3.66 | 1.12 | 4.00 | 2.00 | 1–5 | -0.41 | -0.70 |
| | Item 3 | 121 | 3.89 | 1.15 | 4.00 | 2.00 | 1–5 | -0.80 | -0.37 |
| | Item 4 | 121 | 4.35 | 0.79 | 5.00 | 1.00 | 2–5 | -0.90 | -0.19 |
| | Item 5 | 121 | 4.53 | 0.83 | 5.00 | 1.00 | 1–5 | -2.13 | 4.92 |
| | Item 6 | 121 | 4.34 | 0.93 | 5.00 | 1.00 | 1–5 | -1.41 | 1.63 |
| | Item 7 | 121 | 3.19 | 1.62 | 3.00 | 4.00 | 1–5 | -0.22 | -1.55 |
| | Item 8 | 121 | 4.19 | 1.13 | 5.00 | 1.00 | 1–5 | -1.36 | 1.06 |
| | Item 9 | 121 | 3.55 | 1.55 | 4.00 | 3.00 | 1–5 | -0.60 | -1.18 |
| Ecuador | Item 1 | 100 | 4.64 | 0.59 | 5.00 | 1.00 | 3–5 | -1.42 | 0.96 |
| | Item 2 | 100 | 3.85 | 1.10 | 4.00 | 2.00 | 1–5 | -0.67 | -0.33 |
| | Item 3 | 100 | 4.00 | 1.09 | 4.00 | 1.25 | 1–5 | -1.08 | 0.52 |
| | Item 4 | 100 | 4.28 | 0.96 | 5.00 | 1.00 | 1–5 | -1.67 | 2.71 |
| | Item 5 | 100 | 4.20 | 0.96 | 4.00 | 1.00 | 1–5 | -1.16 | 0.96 |
| | Item 6 | 100 | 4.53 | 0.74 | 5.00 | 1.00 | 2–5 | -1.50 | 1.52 |
| | Item 7 | 100 | 3.54 | 1.39 | 4.00 | 2.00 | 1–5 | -0.62 | -0.85 |
| | Item 8 | 100 | 4.55 | 0.78 | 5.00 | 1.00 | 1–5 | -1.81 | 3.30 |
| | Item 9 | 100 | 3.27 | 1.60 | 4.00 | 3.00 | 1–5 | -0.25 | -1.53 |

**Table S3 (continued)**

*Panel D. Inter-Item Correlations*

| Item | 1 | 2 | 3 | 4 | 5 | 6 | 7 | 8 | 9 |
|---|---|---|---|---|---|---|---|---|---|
| Item 1 | — | .124 | .198 | .222 | .208 | .081 | -.070 | .042 | .013 |
| Item 2 | .082 | — | .536 | .334 | .120 | .056 | -.118 | .053 | -.065 |
| Item 3 | .127 | .467 | — | .409 | .228 | .064 | -.131 | .070 | -.111 |
| Item 4 | .140 | .252 | .325 | — | .347 | .081 | -.129 | .006 | -.041 |
| Item 5 | .128 | .066 | .147 | .224 | — | .097 | -.039 | -.035 | .001 |
| Item 6 | .024 | .020 | .015 | .015 | .025 | — | .134 | .156 | .106 |
| Item 7 | -.070 | -.105 | -.133 | -.125 | -.044 | .073 | — | .186 | .223 |
| Item 8 | .003 | .014 | .022 | -.035 | -.062 | .051 | .113 | — | .095 |
| Item 9 | -.008 | -.082 | -.115 | -.065 | -.017 | .068 | .167 | .055 | — |

*Note.* N = 1,248 participants with probable IGD, defined by an IGDS9-SF total score ≥ 32. Items were rated from 1 (never) to 5 (very often); all item-level data were complete. Mdn = median; IQR = interquartile range; $g_1$ = uncorrected moment skewness; $g_2$ = excess kurtosis. MLR CFA estimated with semTools::compRelSEM. Values are rounded for presentation; analyses used unrounded values.

**Table S4**

*Full-Sample Sensitivity Analyses: Confirmatory Factor Analysis and Measurement Invariance of the IGDS9-SF*

*Panel A. CFA Model Fit in the Full Sample and by Country*

| Sample | Model | Robust χ² | df | p | Robust CFI | Robust TLI | Robust RMSEA [90% CI] | SRMR | AIC | BIC |
|---|---|---|---|---|---|---|---|---|---|---|
| Overall (N = 10,151) | M1 | 2234.043 | 27 | < .001 | .915 | .887 | .099 [.095, .102] | .050 | 275835.4 | 276030.5 |
| | M2 | 1608.503 | 26 | < .001 | .940 | .917 | .085 [.082, .089] | .043 | 275062.4 | 275264.7 |
| | M3 | 1190.156 | 19 | < .001 | .951 | .927 | .086 [.082, .091] | .041 | 240265.4 | 240446.0 |
| Mexico (n = 5,095) | M1 | 1093.529 | 27 | < .001 | .917 | .889 | .097 [.092, .102] | .051 | 139254.7 | 139431.2 |
| | M2 | 828.999 | 26 | < .001 | .938 | .914 | .085 [.080, .090] | .044 | 138927.9 | 139110.9 |
| | M3 | 630.010 | 19 | < .001 | .948 | .924 | .088 [.082, .094] | .042 | 121349.1 | 121512.5 |
| Colombia (n = 2,120) | M1 | 552.000 | 27 | < .001 | .906 | .874 | .105 [.097, .112] | .053 | 58098.5 | 58251.3 |
| | M2 | 379.128 | 26 | < .001 | .936 | .912 | .087 [.080, .095] | .045 | 57893.6 | 58052.1 |
| | M3 | 271.804 | 19 | < .001 | .950 | .926 | .087 [.079, .097] | .042 | 50587.6 | 50729.1 |
| Peru (n = 1,037) | M1 | 279.191 | 27 | < .001 | .907 | .877 | .104 [.093, .115] | .055 | 27227.3 | 27360.8 |
| | M2 | 226.447 | 26 | < .001 | .927 | .899 | .094 [.083, .106] | .049 | 27164.4 | 27302.8 |
| | M3 | 142.491 | 19 | < .001 | .950 | .926 | .088 [.074, .101] | .043 | 23719.8 | 23843.4 |
| Chile (n = 1,276) | M1 | 255.144 | 27 | < .001 | .928 | .905 | .092 [.081, .102] | .045 | 33805.6 | 33944.6 |
| | M2 | 160.088 | 26 | < .001 | .958 | .943 | .071 [.061, .082] | .039 | 33684.4 | 33828.7 |
| | M3 | 119.132 | 19 | < .001 | .966 | .949 | .073 [.061, .086] | .037 | 29320.8 | 29449.6 |
| Ecuador (n = 623) | M1 | 162.391 | 27 | < .001 | .920 | .893 | .099 [.085, .114] | .050 | 17105.6 | 17225.3 |
| | M2 | 121.438 | 26 | < .001 | .944 | .922 | .084 [.070, .100] | .043 | 17056.6 | 17180.8 |
| | M3 | 101.480 | 19 | < .001 | .946 | .920 | .093 [.075, .111] | .045 | 14942.1 | 15052.9 |

**Table S4 (continued)**

*Panel B. Full-Sample Measurement Invariance for Model 3*

| Grouping | Level | Robust χ² | df | p | Robust CFI | Robust TLI | Robust RMSEA [90% CI] | SRMR | Robust Δχ² (Δdf) | pΔ | ΔCFI | ΔRMSEA |
|---|---|---|---|---|---|---|---|---|---|---|---|---|
| Country | Configural | 1253.024 | 95 | < .001 | .951 | .927 | .086 [.082, .090] | .042 | — | — | — | — |
| | Metric | 1333.946 | 123 | < .001 | .950 | .943 | .076 [.073, .080] | .045 | 50.113 (28) | .006 | -.001 | -.010 |
| | Scalar | 1546.447 | 151 | < .001 | .944 | .948 | .073 [.070, .076] | .048 | 198.072 (28) | < .001 | -.006 | -.004 |
| | Strict | 1686.597 | 183 | < .001 | .939 | .953 | .069 [.066, .072] | .053 | 148.798 (32) | < .001 | -.005 | -.004 |
| Sex | Configural | 1227.722 | 38 | < .001 | .950 | .927 | .087 [.083, .091] | .041 | — | — | — | — |
| | Metric | 1255.118 | 45 | < .001 | .950 | .938 | .080 [.076, .084] | .042 | 14.417 (7) | .044 | .000 | -.007 |
| | Scalar | 1334.826 | 52 | < .001 | .948 | .944 | .076 [.072, .079] | .043 | 59.356 (7) | < .001 | -.002 | -.004 |
| | Strict | 1349.931 | 60 | < .001 | .947 | .951 | .071 [.068, .075] | .044 | 29.336 (8) | < .001 | -.001 | -.005 |
| Server | Configural | 1193.440 | 38 | < .001 | .951 | .927 | .086 [.082, .091] | .041 | — | — | — | — |
| | Metric | 1215.407 | 45 | < .001 | .950 | .938 | .080 [.076, .083] | .042 | 10.477 (7) | .163 | .000 | -.007 |
| | Scalar | 1298.799 | 52 | < .001 | .949 | .945 | .075 [.072, .079] | .043 | 63.472 (7) | < .001 | -.002 | -.004 |
| | Strict | 1347.617 | 60 | < .001 | .946 | .950 | .072 [.069, .075] | .045 | 62.962 (8) | < .001 | -.003 | -.003 |
| Game mode | Configural | 1232.264 | 57 | < .001 | .950 | .927 | .087 [.082, .091] | .041 | — | — | — | — |
| | Metric | 1299.154 | 71 | < .001 | .949 | .940 | .079 [.075, .082] | .045 | 47.597 (14) | < .001 | -.001 | -.008 |
| | Scalar | 1484.830 | 85 | < .001 | .944 | .944 | .076 [.072, .079] | .048 | 173.900 (14) | < .001 | -.006 | -.003 |
| | Strict | 1574.928 | 101 | < .001 | .940 | .950 | .072 [.069, .075] | .052 | 102.580 (16) | < .001 | -.004 | -.004 |

*Note.* Full sample N = 10,151. Group sizes were: countries—Mexico=5095; Chile=1276; Colombia=2120; Peru=1037; Ecuador=623; sex—Male=8115; Female=2036; server—LAN=8435; LAS=1716; preferred game mode—Mode 1=3063; Mode 2=4649; Mode 3=2439. M1 = one-factor model with all nine items; M2 = M1 plus the residual covariance between Items 2 and 3; M3 = one-factor model without Item 8 and with the Item 2–Item 3 residual covariance. $\chi^2$, CFI, TLI, and RMSEA are robust MLR indices; RMSEA confidence intervals are 90%. SRMR = standardized root mean square residual; AIC = Akaike information criterion; BIC = Bayesian information criterion. Robust nested comparisons used the Satorra–Bentler scaled difference test (2001 method). ΔCFI and ΔRMSEA were computed from unrounded robust values between consecutive models (metric − configural, scalar − metric, strict − scalar). Invariance change criteria were $\Delta CFI \geq -.010$ and $\Delta RMSEA \leq .015$; all four grouping variables met these criteria through strict invariance. Configural RMSEA was approximately .086 across groupings and should be retained as an absolute-fit caveat. AIC and BIC may be compared between M1 and M2, but not with M3 because M3 uses a different observed-variable set. Values are rounded for presentation; analyses used unrounded values.
